# Multi-parametric MRI-based machine learning model for prediction of WHO grading in patients with meningiomas

**DOI:** 10.1007/s00330-023-10252-8

**Published:** 2023-10-09

**Authors:** Zhen Zhao, Chuansheng Nie, Lei Zhao, Dongdong Xiao, Jianglin Zheng, Hao Zhang, Pengfei Yan, Xiaobing Jiang, Hongyang Zhao

**Affiliations:** 1 Department of Neurosurgery, Union Hospital, Tongji Medical College, Huazhong University of Science and Technology, Wuhan, China; 2 International Education College of Henan University, Kaifeng, China; 3 Department of Geriatric Medicine, Union Hospital, Tongji Medical College, Huazhong University of Science and Technology, Wuhan, China

**Keywords:** Meningioma, WHO grading, Radiomics, Machine learning, Nomogram

## Abstract

**Objective:**

The purpose of this study was to develop and validate a nomogram combined multiparametric MRI and clinical indicators for identifying the WHO grade of meningioma.

**Materials and methods:**

Five hundred and sixty-eight patients were included in this study, who were diagnosed pathologically as having meningiomas. Firstly, radiomics features were extracted from CE-T1, T2, and 1-cm-thick tumor-to-brain interface (BTI) images. Then, difference analysis and the least absolute shrinkage and selection operator were orderly used to select the most representative features. Next, the support vector machine algorithm was conducted to predict the WHO grade of meningioma. Furthermore, a nomogram incorporated radiomics features and valuable clinical indicators was constructed by logistic regression. The performance of the nomogram was assessed by calibration and clinical effectiveness, as well as internal validation.

**Results:**

Peritumoral edema volume and gender are independent risk factors for predicting meningioma grade. The multiparametric MRI features incorporating CE-T1, T2, and BTI features showed the higher performance for prediction of meningioma grade with a pooled AUC = 0.885 (95% CI, 0.821–0.946) and 0.860 (95% CI, 0.788–0.923) in the training and test groups, respectively. Then, a nomogram with a pooled AUC = 0.912 (95% CI, 0.876–0.961), combined radiomics score, peritumoral edema volume, and gender improved diagnostic performance compared to radiomics model or clinical model and showed good calibration as the true results. Moreover, decision curve analysis demonstrated satisfactory clinical effectiveness of the proposed nomogram.

**Conclusions:**

A novel nomogram is simple yet effective in differentiating WHO grades of meningioma and thus can be used in patients with meningiomas.

**Clinical relevance statement:**

We proposed a nomogram that included clinical indicators and multi-parameter radiomics features, which can accurately, objectively, and non-invasively differentiate WHO grading of meningioma and thus can be used in clinical work.

**Key Points:**

• *The study combined radiomics features and clinical indicators for objectively predicting the meningioma grade*.

• *The model with CE-T1* + *T2* + *brain-to-tumor interface features demonstrated the best predictive performance by investigating seven different radiomics models*.

• *The nomogram potentially has clinical applications in distinguishing high-grade and low-grade meningiomas*.

**Supplementary Information:**

The online version contains supplementary material available at 10.1007/s00330-023-10252-8.

## Introduction

Meningiomas are the most common primary benign intracranial tumors, accounting for approximately 36.1% of central nervous system tumors. The annual worldwide incidence ranges from 50.4 to 76.1 per 1,000,000 people [1, 2]. Patients with meningiomas are more common in women than men and increase with age. Although meningiomas are generally considered benign tumors, a subset shows aggressive biological and clinical behaviors. According to the CBTRUS report in 2021, high-grade meningiomas (WHO grade II and III) accounted for 19.9% of all newly diagnosed meningiomas between 2014 and 2018 [3]. High-grade meningiomas have been associated with high recurrence and poor prognosis, with a 30–50% higher recurrence rate than that of low-grade meningiomas during a 10-year follow-up [4, 5]. Additionally, surgical protocols and postoperative treatments differ significantly between high- and low-grade meningiomas. Hence, preoperative knowledge of the meningioma grade is clinically important.

To date, radiological examinations remain the crucial modalities for the preoperative diagnosis of meningiomas. In particular, magnetic resonance imaging (MRI), with the advantages of good tissue contrast, no bone artifacts, and multi-faceted imaging [6], was currently the preferred imaging protocol for the preoperative diagnosis and the differential diagnosis between the high-grade and low-grade meningiomas [7]. Studies have revealed different degrees of differences in conventional MRI imaging features, such as tumor location, shape, size, brain–tumor interface (BTI), tumor necrosis, heterogeneous tumor enhancement, and peritumoral edema volume (PEV), between high-grade and low-grade meningiomas [8–11]. Advanced imaging technology, including diffusion-weighted MRI imaging and positron emission tomography, is also promising [12, 13], whereas the assessed features were marked subjectively and qualitatively in these studies, which highly relied on the experience and expertise knowledge of the neurosurgeons or neuroradiologist, and thus limit the use in clinical practice. Therefore, objective and quantitative methods may be more preferable in these scenarios.

Radiomics is an emerging imaging analysis method that may overcome these limitations [14]. It can extract multidimensional features by applying powerful computer image-processing capabilities and multiple big-data mining methods. Although these features are difficult to acquire visually, they preserve research-related information such as first-order, shape, texture, and filter-transformed features from digitally encrypted medical images (CT, MRI, and PET) [15, 16]. These objective features can accurately quantify the phenotypic and provide underlying pathophysiological information of the regions of interest (ROI) in the images, while avoiding the subjective differences caused by manual reading [17]. There have been many studies reported the application of radiomics in intracranial tumors, such as for predicting tumor type [6, 18], tumor grade [19], histological subtype [20, 21], and the recurrence-free survival for patients with malignant tumors [22]. Likewise, blood indicators are diagnostic markers for noninvasive grading of meningiomas [23, 24]. To our knowledge, the meningioma grade has only been predicted using clinical features, blood markers, or single-parameter MRI. Zhang et al [25] found that among different models, a clinical radiomics model integrating MRI sequences and sex had the best predictive ability for brain invasion. Surely, PET might also be useful for noninvasive prediction of the meningioma grade. A meta-analysis conducted K Mariam et al explored the role of PET in meningioma grading. The pooled data showed standardized uptake value and tumor-to-normal in [^18^F]FDG PET can be useful to noninvasively differentiate benign from malignant meningiomas [26]. Even so, meningioma is the benign tumor; the patients can be diagnosed by general MRI instead of expensive PET. So far, few studies have evaluated and compared multiomics models for predicting the meningioma grade.

Thus, this study aimed to evaluate the diagnostic performance of a multiparametric MRI model and develop a nomogram for clinical application to distinguish between high-grade and low-grade meningiomas.

## Materials and methods

### Study population

This retrospective study was conducted in accordance with the Declaration of Helsinki and approved by the institutional review board of Wuhan Union Hospital; the patients’ informed consent was waived owing to the retrospective nature of the study. We collected patients who were pathologically confirmed as meningiomas from January 2012 to December 2020.

**Fig. 1:**
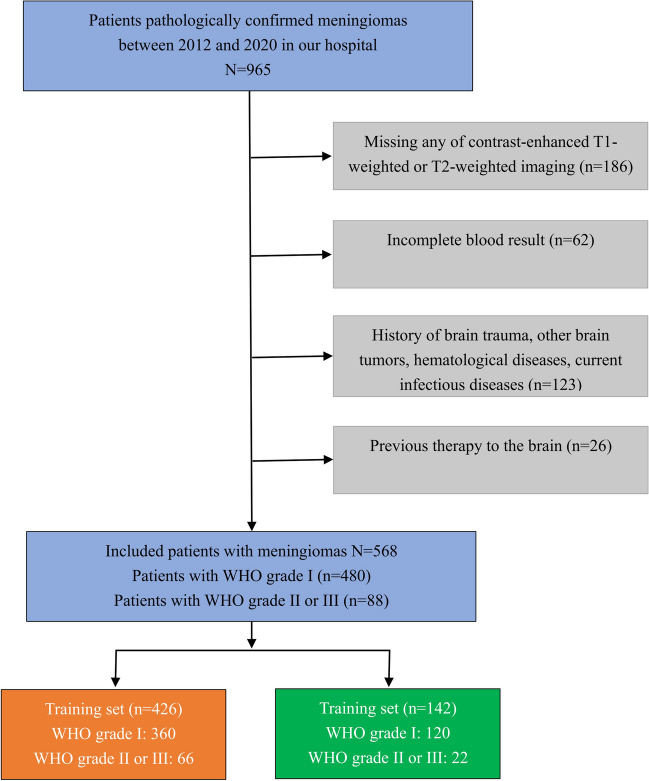
The flowchart of patient selection

All patients were enrolled by the inclusion and exclusion criteria. The inclusion criteria were as follows: (a) patients were pathologically diagnosed with meningioma and clearly graded by histology; (b) preoperative MRI included CE-T1 and T2 sequences; (c) the number of lesion-bearing image slices was not less than three; (d) blood examinations, including blood routine test and liver function test, were performed within 2 weeks before surgery; (e) there were no apparent signs of infection. The exclusion criteria were as follows: (a) incomplete MRI data; (b) with the history of brain trauma, brain tumors, surgery, hematological diseases, or ongoing infectious diseases; (c) have received chemotherapy, radiotherapy, or hormone therapy for any reasons before surgery. The patient recruitment flowchart is presented in Fig. 1.

Baseline clinicopathologic data, including age, gender, and preoperative WHO grade, were derived from the hospital electronic medical record system. Routine blood and liver function tests within 2 weeks before surgery were also obtained. If multiple results were available, the latest results before operation were included, including the absolute counts of white blood cells (WBCs), red blood cells, platelets, neutrophils, lymphocytes, and monocytes and levels of albumin, fibrinogen, and hemoglobin.

The following blood indices were calculated, as shown in Supplementary Material 1: neutrophil-to-lymphocyte ratio (NLR), derived NLR, platelet-to-lymphocyte ratio, monocyte-to-lymphocyte ratio, lymphocyte-to-monocyte ratio, neutrophil-to-platelet ratio, NPI, and SII. These indicators are biomarkers for the differential diagnosis or prognosis prediction of many intracranial tumors [27–29].

### MRI acquisition and segmentation

The MRIs including contrast-enhanced T1-weighted (CE-T1) images and T2-weighted (T2) images were acquired from the PACS system in our hospital. These images were performed using a 1.5-T or 3.0-T magnetic resonance clinical scanner with standard head and neck coils, and the scans were performed in coronal, sagittal, and transverse positions. The imaging protocols are shown in Supplementary Material 2. The CE-T1 images were acquired within 200 s after injection of gadopentetate dimeglumine or other gadolinium products (0.2 mmol/kg). The CE-T1 and T2 images were used for subsequent analysis.

The ROIs were manually drawn slice by slice on the CE-T1 and T2 images for all patients using the open-source ITK-SNAP software (University of Pennsylvania, www.itksnap.org). The tumor was manually segmented on CE-T1 images by the first researcher (D.D.X.). The second researcher (Z.Z.) similarly manually drew peritumoral edema on the T2 images in the same way. As this region also included the tumor, the peritumoral edema volume (PEV) was measured by deducting the tumor magnitude from the total volume of this outlined region. Similarly, the ROIs of the brain-to-tumor interface (BTI) were also manually drawn by Z.Z. along the tumor outline on CE-T1 images. A 1-cm brush width was set in the ITK-SNAP software to segment along the tumor outline, such that the boundary would expand by 0.5 cm in the inside and outside directions at the tumor outline to form a 1-cm-thick BTI ROI. BTI radiomics features have been explored for predicting meningioma invasion but not for grading meningiomas. To ensure the stability and accuracy of the segmented data, 50 patients were randomly selected from the entire sample after the first segmentation. Subsequently, the two investigators cross-segmented the CE-T1 and T2 images again to assess interobserver reliability and calculate the interobserver correlation coefficient (ICC). If D.D.X. and Z.Z. were unsure about the tumor boundaries, the third researcher P.F.Y. with 10 years of neurosurgery clinical experience confirmed the segmentation. A previous study reported the introduction of irrelevant information when painting a very small tumor area. Therefore, sections with very small tumor areas (< 10 pixels) were eliminated during tumor segmentation in the present study.

### Radiomics feature extraction

The feature extraction process was performed using the open-source Python package called pyradiomics (version 3.0.0, https://github.com/AIM-Harvard/pyradiomics) [30]. To minimize the effects of heterogeneous datasets due to different scanners and MRI protocols, the images were preprocessed prior to feature extraction, including normalization, discretization, and resampling to a 1 × 1 × 1 mm isotropic voxel size. These steps are recommended as part of the workflow by software package developers to increase the reliability and robustness of radiomics analysis [31]. In this study, 1015 features were extracted for each patient from the CE-T1, T2, and BTI images (Table S1). Each feature set included 14 shape features, 198 first-order features, and 803 textural features. Additionally, the pyradiomics package was used to calculate the tumor volume and volume of the edema-outlined area. Detailed information regarding this algorithm is provided in Supplementary Material 3.

### Feature selection method

Radiomics features may have different dimensions, so the differences between feature values may vary widely [32]. The features extracted from the CE-T1, T2, and BTI images were normalized using *z*-scores to eliminate dimension and value range differences between the indicators. The mean value of the processed data was 0, and the standard deviation was 1. The conversion formula is as follows:

$${X}^{*}=\frac{X-\overline{X}}{\sigma }$$

where ${X}^{*}$ is the transformed eigenvalue of the variable X, $\overline{X }$ is the mean value of the original data, and $\upsigma$ is the standard deviation of the original data.

The included patients were divided into training and test groups in a ratio of 3:1 through stratified random sampling. High-dimensional features may contain irrelevant or highly redundant data, which may prolong the model training time, cause model overfitting, and greatly reduce the performance of machine learning classifiers. Therefore, feature selection is an indispensable step for machine learning. In this study, the CE-T1, T2, BTI, CE-T1 + T2, CE-T1 + BTI, T2 + BTI, and CE-T1 + T2 + BTI imaging features were analyzed using the variance selection method, and features with *p* < 0.05 were selected. Subsequently, least absolute shrinkage and selection operator (LASSO) regression, which is especially suitable for small sample sizes with large variables, was performed to select the most predictive features with non-zero coefficients that could predict high-grade meningioma from the training groups of CE-T1, T2, BTI, CE-T1 + T2, CE-T1 + BTI, T2 + BTI, and CE-T1 + T2 + BTI. The fivefold cross-validation with recursive feature elimination method was performed to explore the value of *λ* corresponding to the lowest partial likelihood deviance. The mathematical formula of LASSO was showed in the Supplementary Material 4. When *λ* is large, the feature coefficients are compressed. When *λ* reaches a certain value, a few unimportant variables are compressed to 0, indicating that the feature has been eliminated from the model. In Figure S1, the curve falling from left to right is gradually compressed by the increasing *λ* until it is 0 in the left figure. Hence, the *λ* value with the lowest average error was chosen through fivefold cross-validation to filter the characteristics in the right figure. The radiomics score was calculated for each patient using a linear combination of the selected features weighted by their corresponding coefficients in all models.

### Construction of the classification models

There were some deviations from the classification results when the unbalanced datasets were trained using a machine-learning classifier. Moreover, the performance of the classifier may be unrealistically evaluated if the validation dataset is unbalanced. Thus, we applied the synthetic minority oversampling technique (SMOTE) to avoid the above problem [33]. The support vector machine (SVM) algorithm was used to build radiomics models with a radial basis kernel for risk prediction of high-grade meningiomas. The maximum area under the curve (AUC) with fivefold cross-validation was used to determine the optimal value of the regularization parameter in the training group. Additionally, the AUC, accuracy, sensitivity, specificity, positive predictive value (PPV), and negative predictive value (NPV) were calculated to evaluate the performance of the radiomics model for quantitatively predicting high- and low-grade meningiomas in both the training and test groups. The CE-T1, T2, and BTI models were built based on CE-T1, T2, and BTI features, respectively. Furthermore, the combined models were also built based on the different combinations of CE-T1, T2, and BTI features, including CE-T1 + T2, CE-T1 + BTI, T2 + BTI, and CE-T1 + T2 + BTI models.

### Development and validation of a nomogram

Stepwise logistic regression was conducted to seek significant clinical predictors in the training group with the following clinical indicators: age, gender, tumor volume, PEV, and blood indicators. The Wald test was applied to calculate *p*-values for each indicator.

After radiomics model comparison and clinical indicator selection, an individualized nomogram containing radiomics score and meaningful clinical predictors was developed in the training group and validated in the test group.

**Fig. 2:**
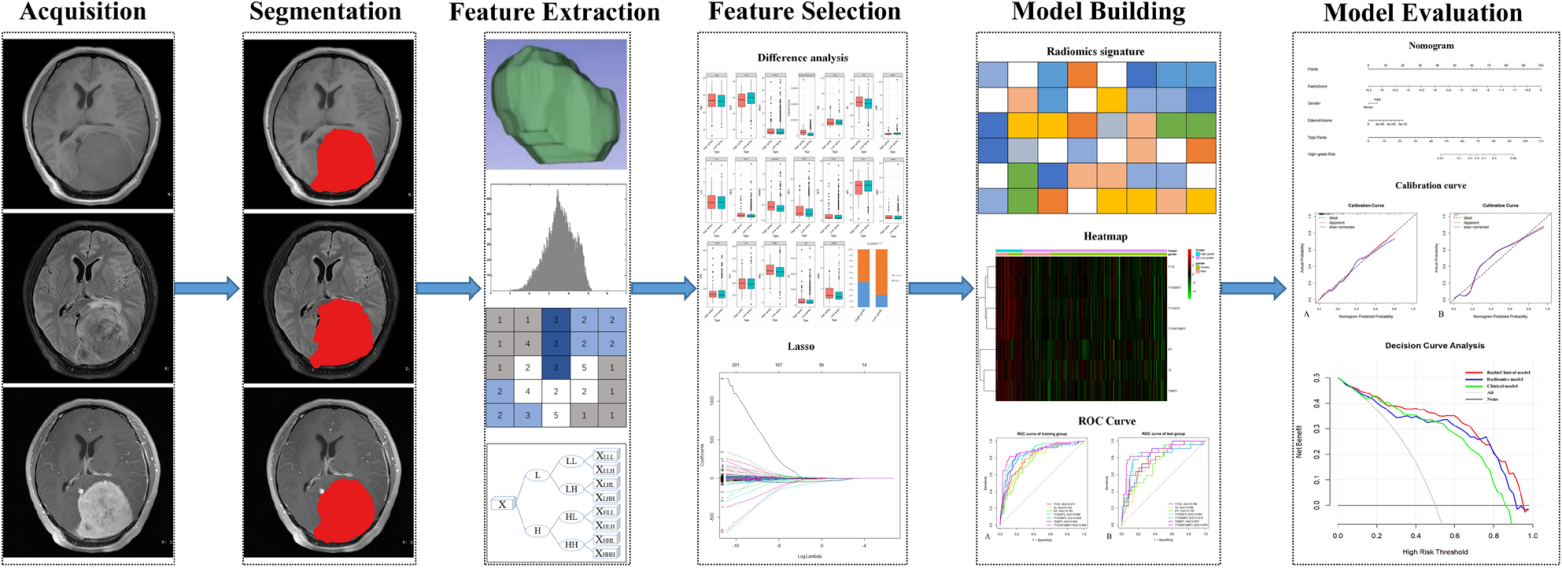
The overall workflow of radiomics processing and nomogram construction

The discriminative performance of nomogram was assessed using calibration curves in the training and test groups, and the Hosmer–Lemeshow test was used to assess the consistency between the predicted risk of high-grade meningioma and the observed outcomes. Additionally, decision curve analysis (DCA) was performed to evaluate the clinical utility of the nomogram compared with that of other models at different threshold probabilities. Figure 2 shows an overall flowchart of the study.

### Statistical analysis

Statistical analyses and graphical visualization were achieved by using Python (version 3.7, http://www.python.org) and R (version 4.0.1; http://www.R-project.org). Python was used to complete imaging normalization and radiomics feature extraction. While R was used to select the radiomics features, construct and evaluate the prediction models and compare the clinical indicators between low- and high-grade meningiomas. Continuous variables are displayed as means ± standard deviations or medians and interquartile ranges, whereas categorical variables are presented as absolute and relative frequencies. The unpaired *t*-test or nonparametric test with the Mann–Whitney *U* test was used for descriptive comparisons of continuous variables between the high-grade and low-grade meningioma groups, whereas the chi-square test or Fisher exact test was used for descriptive comparison of categorical variables between the high-grade and low-grade meningioma groups. All statistical tests were two-tailed and conducted with the statistical significance level set at *p* < 0.05.

## Results

### Patient characteristics

Overall, 568 patients were included, of whom 480 with low-grade meningiomas (including 480 WHO grade I patients) and 88 with high-grade meningiomas (including 79 WHO grade II and 9 WHO grade III patients). The mean age was 52.8 years, and 434 patients were women. Among them, 426 (54.3 ± 9.8 years, 334 women) and 142 (52.6 ± 11.3 years, 100 women) patients were assigned to the training and independent test groups, respectively. The clinical characteristics of the training and test groups are shown in Table 1.

**Table 1:** Baseline characteristics of patients with meningiomas

	Low-grade meningiomas	High-grade meningiomas	*p* value
*N*	480	88	
Age (mean ± SD)	53.2 ± 9.7	53.6 ± 12.17	0.893
Gender (%)			** < 0.001**
Male	378	40	
Female	98	52	
TV (median [IQR])	9823.26 [4949.48, 18,792.47]	10,828.50 [6044.23, 21,169.28]	0.088
PEV (median [IQR])	13,284.73 [4044.704, 45,226.822]	43,085.56 [15,049.209, 95,174.959]	** < 0.001**
Laboratory test (median [IQR])			
RBC	4.21 [3.840, 4.520]	4.28 [4.020, 4.690]	0.153
Hemoglobin	125 [115, 135]	128 [119, 139]	0.060
Platelet	192 [172, 252]	198 [166, 276]	0.791
WBC	5.99 [4.84, 8.470]	6.60 [5.33, 10.17]	**0.038**
Neutrophil	3.70 [2.63, 6.28]	3.99 [3.06, 7.40]	0.066
Lymphocyte	1.53 [1.04, 1.93]	1.49 [1.08, 2.00]	0.855
Monocyte	0.37 [0.28, 0.48]	0.44 [0.32, 0.61]	**0.003**
Albumin	40.10 [36.30, 42.90]	38.90 [34.40, 42.10]	0.072
FIB	2.91 [2.540, 3.320]	2.82 [2.460, 3.450]	0.718
NLR	2.20 [1.546, 5.157]	2.56 [1.722, 5.727]	0.196
dNLR	1.66 [1.211, 3.313]	1.86 [1.312, 3.128]	0.265
PLR	130.6 [100.0, 180.3]	135.8 [107.3, 172.5]	0.936
MLR	0.244 [0.172, 0.380]	0.252 [0.208, 0.408]	0.086
LMR	4.095 [2.857, 5.829]	3.697 [2.391, 4.800]	0.063
NPR	0.018 [0.013, 0.035]	0.022 [0.016, 0.037]	0.061
NPI	48.10 [43.30, 51.75]	48.00 [40.60, 51.25]	0.304
SII	449.7 [292.9, 939.7]	517.6 [342.2, 1094.5]	0.217

*SD*, standard deviation; *TV*, tumor volume; *PEV*, peritumoral edema volume; *IQR*, interquartile range; *RBC*, red blood cell; *WBC*, white blood cell; *FIB*, fibrinogen; *NLR*, neutrophil-to-lymphocyte ratio; *NPR*, neutrophil-to-platelet ratio; *dNLR*, derived neutrophil-to-lymphocyte ratio; *LMR*, lymphocyte-to-monocyte ratio; *MLR*, monocyte-to-lymphocyte ratio; *PLR*, platelet-to-lymphocyte ratio; *NPI*, prognostic nutritional index; *SII*, systemic immune inflammatory index. The bold entries represented the statistically significant differences between high and low grade meningiomas

The analyses of clinical indicators showed that gender, peritumoral edema volume, white blood cell count, and monocyte count were found to be significantly different statistically between the high-grade and low-grade groups in both training and test groups. The patients with high-grade meningiomas had a higher proportion of males (*p* < 0.001) and larger peritumoral edema volume (*p* < 0.001) than in those with low-grade meningiomas. Furthermore, the higher count of white blood cell and monocyte was showed in patients with high-grade meningiomas than with low-grade meningiomas. While with tumor volume, blood indices showed no significant difference between the two groups. The differential distributions of clinical indicators in high-grade and low-grade meningiomas are exhibited in Figure S2.

### Feature selection

Satisfactory interobserver reproducibility was achieved for CE-T1, T2, and BTI imaging features. The ICCs of the CE-T1, T2, and BTI features were 0.923 (95% confidence interval (CI), 0.865–0.978), 0.881 (95% CI, 0.836–0.952), and 0.909 (95% CI, 0.882–0.965), respectively.

In the radiomics features, based on the variance selection method, the number of CE-T1 image features was reduced from 1015 to 451; T2 image features from 1015 to 569; BTI features from 1015 to 328; CE-T1 + T2 image features from 2030 to 1020; CE-T1 + BTI image features from 2030 to 779; T2 + BTI image features from 2030 to 879; CE-T1 + T2 + BTI image features from 3045 to 1348 in the training group. Then, LASSO regression was conducted to select the most representative radiomics features. The features included of every region through LASSO regression are added in the Table S2. Following this, multivariate logistic regression was used to remove non-identifiable radiomics features. Finally, the radiomics scores of each patient were computed according to the filtered radiomic features and corresponding coefficients in the training and test groups. The radiomics scores of the training and test groups are supplemented in Tables S3 and S4. Figure S3 is a heatmap showing the distribution of the radiomics scores in the seven radiomics feature sets.

### Construction and validation classification models

**Fig. 3:**
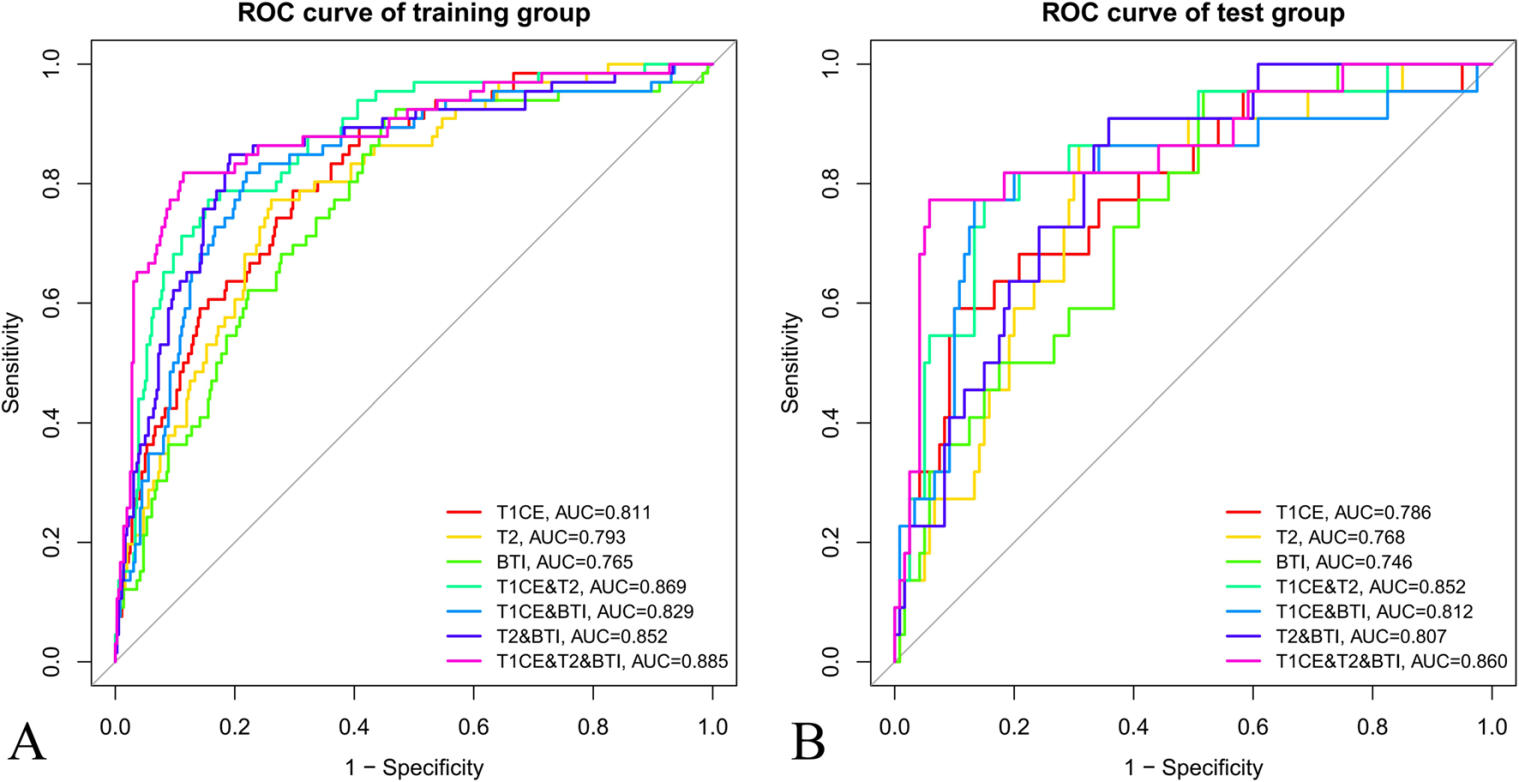
The predictive performance of identifying between high-grade and low-grade meningiomas in different radiomics models. **A** The receiver operating characteristic curve (ROC) and the area under the curve (AUC) of seven different radiomics are showed in the training group, respectively. **B** The ROC and AUC of seven different radiomics are showed in the test group, respectively. The radiomics features based on CE-T1 + T2 + BTI showed the best predictive performance in both the training group and test group

The discriminative ability of the SVM model was firstly evaluated in the training group and then independently validated in the test group. The AUC, sensitivity, specificity, PPV, and NPV of the CE-T1, T2, BTI, CE-T1 + T2, CE-T1 + BTI, T2 + BTI, and CE-T1 + T2 + BTI models were calculated (Table 2). Figure 3 depicts the receiver operating characteristic curves of the different models. The CE-T1 + T2 + BTI model showed the best performance in predicting high-grade meningiomas both in the training and test groups, with AUCs of 0.885 (95% CI, 0.821–0.946) and 0.860 (95% CI, 0.788–0.923), respectively. Thus, the multiparametric radiomics model was significantly better than the single-parameter radiomics models in previous studies.

**Table 2:** Diagnostic performance of different radiomics models in the training and test groups

	Training group					Test group				
	AUC (95%CI)	Sensitivity	Specificity	PPV	NPV	AUC (95%CI)	Sensitivity	Specificity	PPV	NPV
CE-T1	0.811 (0.751–0.882)	0.788	0.703	0.726	0.768	0.786 (0.711–0.865)	0.591	0.900	0.855	0.689
T2	0.793 (0.730–0.866)	0.773	0.739	0.748	0.765	0.768 (0.709–0.833)	0.864	0.692	0.737	0.835
BTI	0.765 (0.716–0.826)	0.909	0.550	0.669	0.858	0.746 (0.688–0.818)	0.955	0.483	0.649	0.915
CE-T1 + T2	0.869 (0.796–0.925)	0.773	0.844	0.832	0.788	0.864 (0.789–0.926)	0.773	0.850	0.837	0.789
CE-T1 + BTI	0.829 (0.756–0.893)	0.818	0.781	0.789	0.811	0.812 (0.718–0.906)	0.773	0.867	0.853	0.792
T2 + BTI	0.852 (0.791–0.908)	0.848	0.808	0.815	0.841	0.807 (0.735–0.882)	0.909	0.642	0.717	0.876
CE-T1 + T2 + BTI	0.885 (0.821–0.946)	0.828	0.896	0.889	0.839	0.860 (0.788–0.923)	0.773	0.942	0.930	0.806
Clinical model	0.697 (0.632–0.762)	0.723	0.668	0.685	0.707	0.726 (0.640–0.790)	0.734	0.697	0.707	0.723

*CI*, confidence interval; *CE-T1*, contrast-enhanced T1-weighted; *T2*, T2-weighted; *BTI*, brain-to-tumor interface; *PPV*, positive predictive value; *NPV*, negative predictive value

### Development of an individualized nomogram

**Fig. 4:**
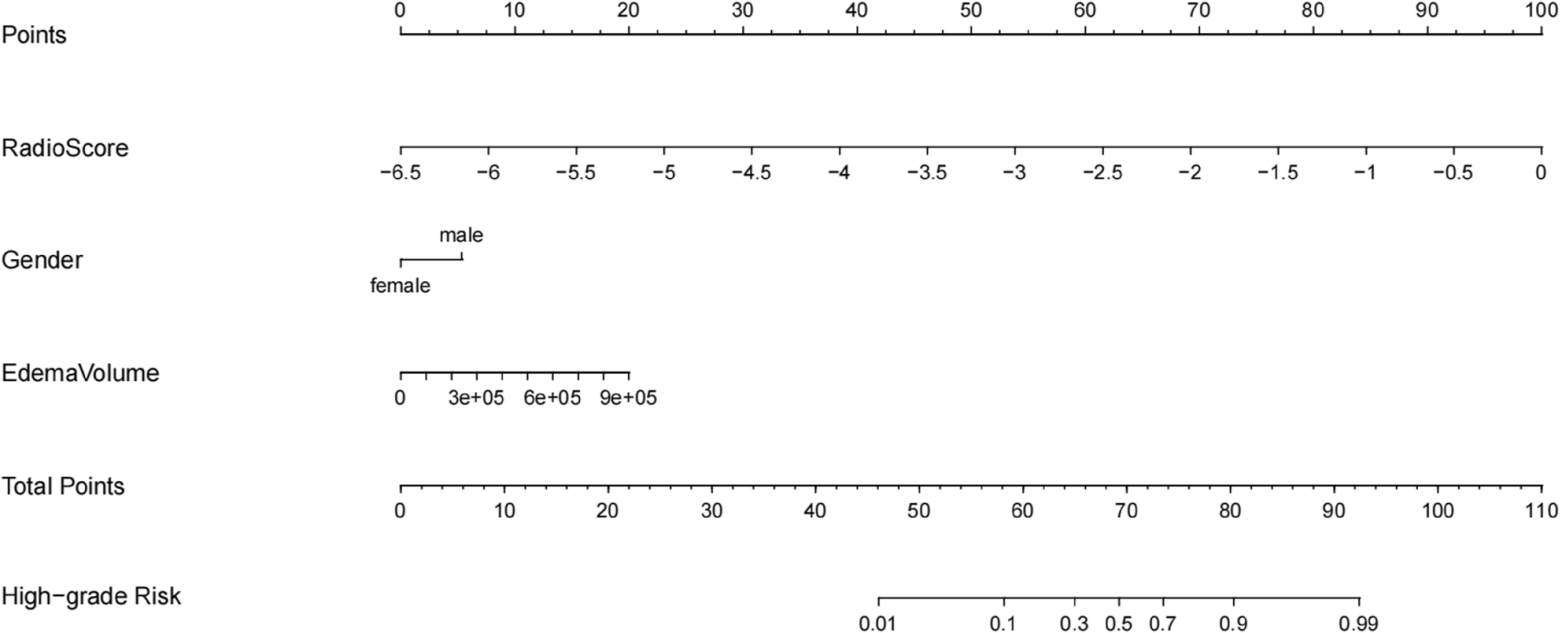
The nomogram was developed with the radioclinical model in the training group. The radiomics score, gender, and peritumoral edema volume were included in the nomogram. The high-grade risk represented the predictive probability of high-grade meningiomas

Based on the radiomics model exploration described above, the features in the optimal model and their corresponding coefficients were used to calculate the radiomics score for each patient. Logistic regression identified the radiomics score, sex, and PEV as independent predictors for discriminating between high-grade and low-grade meningiomas. A model incorporating these independent predictors was developed and presented as a nomogram (Fig. 4).

### Predictive performance evaluation of the nomogram

The individualized nomogram yielded AUCs of 0.912 (95% CI, 0.876–0.961) and 0.896 (95% CI, 0.822–0.946) in the training and test groups, respectively. The nomogram was significantly superior to the radiomics and clinical models in both the training and test groups. In addition, the performance of this nomogram in predicting meningioma grading is significantly higher than that reported by other studies on meningioma grading.

**Fig. 5:**
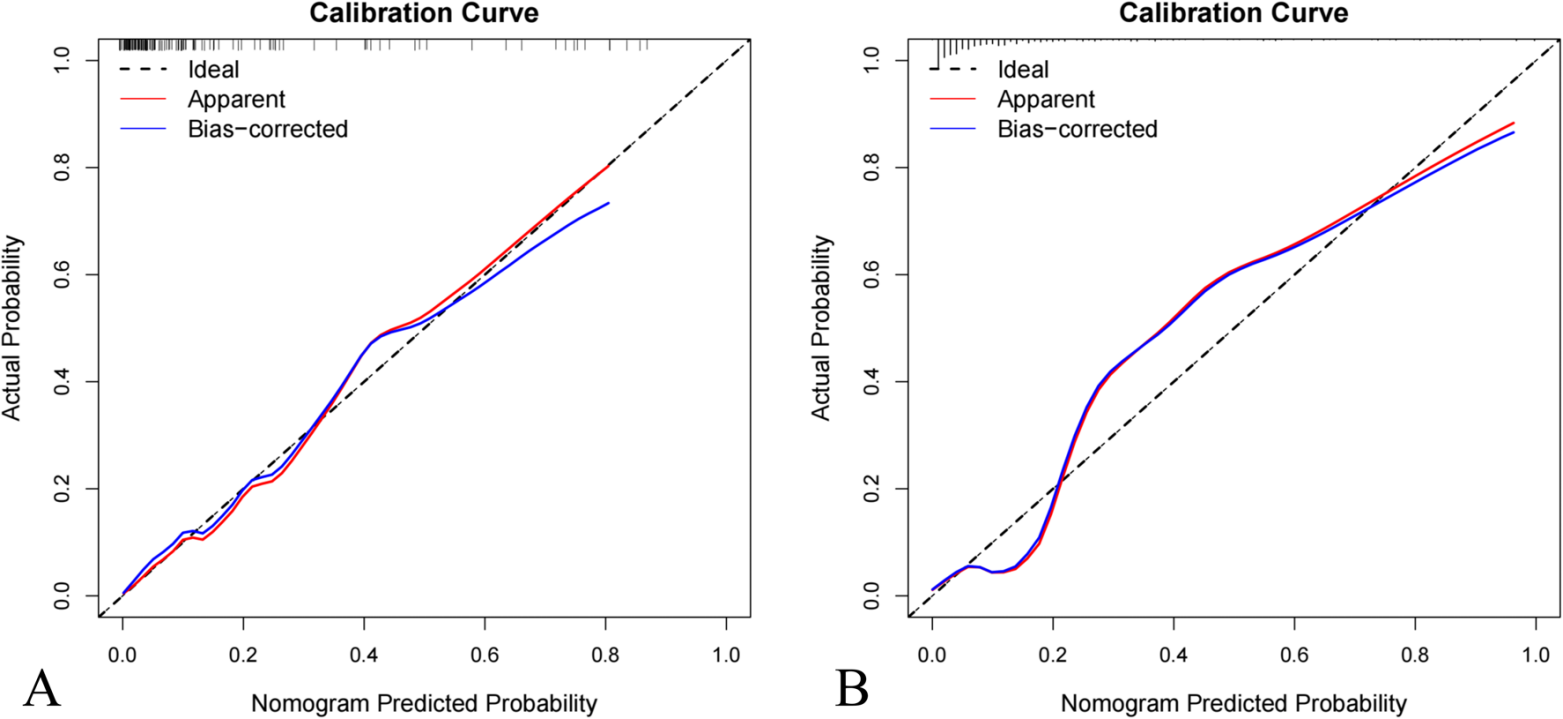
Calibration curves of the nomogram. **A** Calibration curve of the nomogram in the training group. **B** Calibration curve of the nomogram in the test group. The calibration curve showed the calibration of the models in terms of the consistency between the predictive performance of high-grade meningiomas and the actual results observed for calibration. The Hosmer–Lemeshow test showed *p* = 0.629 and *p* = 0.316 in the training and test groups, respectively

Subsequently, the calibration curve of the nomogram demonstrated good calibration in the training and test groups (Fig. 5A and B). The Hosmer–Lemeshow test showed no statistically significant difference between the predicted and actual results in both the training and test groups (*p* = 0.629 and *p* = 0.316, respectively), suggesting that the predictive performance of the nomogram did not deviate perfectly.

**Fig. 6:**
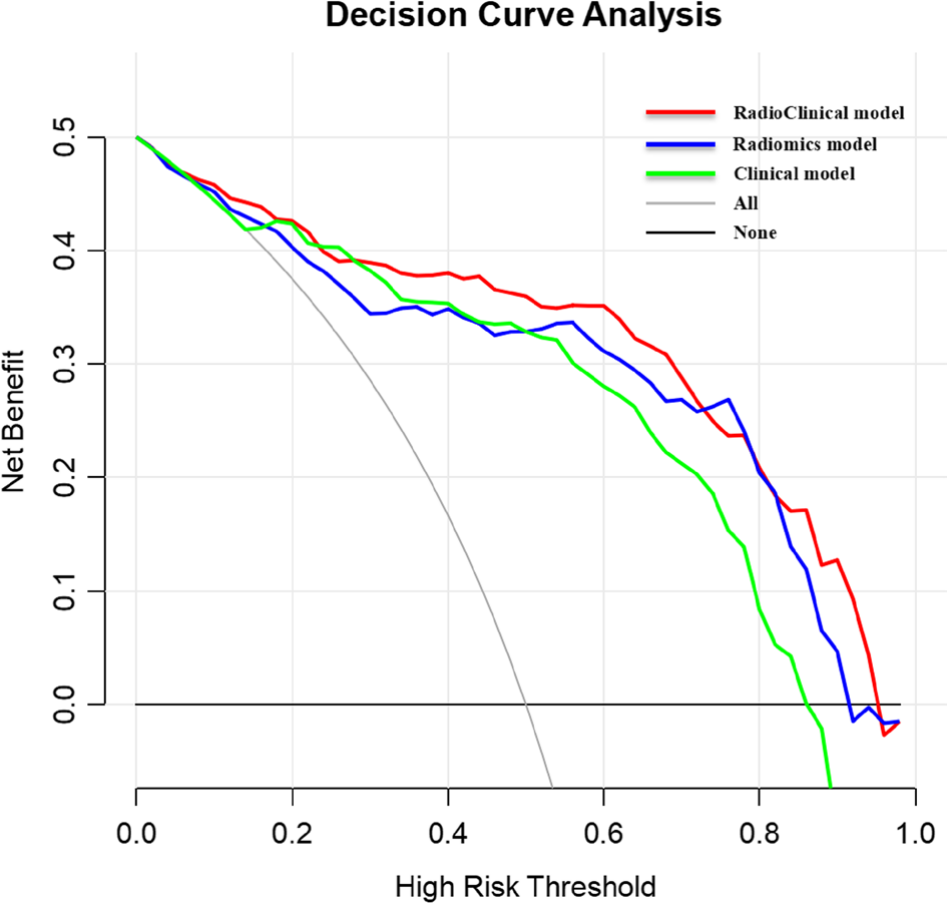
Decision curve analysis for the radioclinical nomogram, radiomics model, and clinical model. The decision curve showed that the radioclinical nomogram was used to differentially diagnose between high-grade and low-grade meningiomas has a greater advantage than used the radiomics model or clinical model in terms of clinical application

Furthermore, we also explored the clinical usefulness of the nomogram. The DCA revealed that the radioclinical nomogram used to predict high-grade meningiomas has a greater advantage than the radiomics or clinical model or treating no patients or all patients in terms of clinical application. The DCA results for the clinical model, radiomics model, and radioclinical nomogram are presented in Fig. 6.

## Discussion

Surgical resection is the primary treatment for patients with meningiomas. Observation or Gamma Knife surgery may be optimally considered for asymptomatic patients with low-grade meningiomas [34], whereas high-grade meningiomas were required surgical resection and a detailed adjuvant radiotherapy protocol [35]. Hence, preoperative knowledge of the meningioma grade is clinically important to patients and neurosurgeons. Studies have predicted the meningioma grade according to qualitative features, such as tumor location, size, and shape, degree of peritumoral edema, and the presence or absence of heterogeneous enhancement. Conversely, radiomics methods based on conventional MRI can quantitatively, non-invasively, and objectively predict the histological grade of meningiomas.

Recently, Coroller et al [36] explored the value of radiomics features in predicting the meningioma grade using CE-T1 images. Furthermore, Park et al [37] and Sacco et al [38] showed that a combination of MRI sequences adds value to tumor grading with homogeneous imaging data from the same scanner. To date, however, studies on meningioma grading have primarily focused on the analysis of internal tumor contents instead of the BTI using radiomics methods. In 2021, Joo et al [39] developed and validated a combined model based on six radiomics features at the BTI to predict brain invasion in meningioma patients. The predictive model showed superior performance in both the training and test groups. Subsequently, Zhang et al [25] confirmed that radiomics of the BTI can accurately predict meningioma aggressiveness. On the one hand, high-grade meningiomas are more aggressive than low-grade meningiomas. On the other hand, the BTI provides some information about the disruption of the meningeal surface. Thus, we speculate that radiomics at the BTI may be promising for predicting the meningioma grade.

In this study, we developed and validated a multiparametric MRI model to predict high-grade and low-grade meningiomas. Seven radiomics models were highly correlated with high-grade meningiomas and remained stable in both the training and test groups. The CE-T1 + T2 + BTI radiomics model demonstrated the best predictive power in both the training (AUC: 0.895) and test (AUC: 0.860) groups. This finding is largely consistent with those reported in previous studies, indicating that the multiparametric radiomics model was better than the single-parameter radiomics model. Meanwhile, the nomogram comprising the radiomics score, PEV, and sex showed the best discriminative power in both the training (AUC: 0.912) and test (AUC: 0.896) groups and could discriminate > 90% high-grade meningiomas.

In our study, the overall incidence of high-grade meningiomas was largely consistent with the 13–24% reported in other larger series. Ultimately, the subgroup analysis based on clinical indicators showed that PEV and gender were the independent predictors of high-grade meningioma. We found that high-grade meningiomas occurred significantly more frequently in male patients, similar to the findings of other studies [40–42]. However, one study had conflicting results, identifying no correlation between the meningioma grade and gender [11]. Furthermore, PEV was also positively correlated with the meningioma grade, and meningiomas with extended peritumoral edema were significantly more frequent than high-grade meningiomas. Peritumoral edema is caused by compressive ischemia of the brain tissue, parasitism of leptomeningeal capillaries, and secretion-excretion phenomena by tumor cells [43]. A previous study found that edema volume was an independent risk factor for meningioma aggressiveness, with an odds ratio of 1.01, which may be why PEVs can predict the meningioma grade [44]. Although age and tumor volume are reportedly strong preoperative predictors of high-grade meningiomas [9], we did not observe this trend. Therefore, these hypotheses need to be further explored in larger studies.

A total of 1015 radiomics features, most of which were wavelet-derived features, were extracted in this study. The wavelet transform can decompose and reconstruct the data at multiple scales to obtain images or data with different scale information for which image data has different spatial resolutions and frequency information. This helps to further extract and analyze useful biological features in images, such as morphology, texture, and blood flow [45]. Therefore, many wavelet-derived features were included to more realistically reflect the grading differences of meningiomas. Feature preprocessing is a necessary procedure in machine learning. Standardization of feature values cannot only transform data of different magnitudes into the same magnitude and make them comparable but also improve the rate of convergence and reduce the amount of computation [32]. In this study, the *z*-score was performed for feature numerical normalization, which is especially useful for the scenarios in which the maximum and minimum values in the data were unknown [6]. High-dimensional data features can lead to model overfitting. To overcome this fatal flaw, variation analysis and LASSO regression were used for feature selection, and the SVM classifier was used to build the final classification model. First, variation analysis was used to screen for features with no significance between high-grade and low-grade meningiomas for follow-up research. As a commonly used automatic feature selection method, LASSO regression can reduce the number of features without significantly increasing bias [46]. The SVM classifier is one of the most powerful algorithms in machine learning and reportedly has good classification performance in predicting meningioma grades [47]. Simultaneously, we also applied fivefold cross-validation to minimize overfitting as much as possible. Furthermore, to overcome the impact of imbalanced datasets on classifier fitting, SMOTE oversampling was performed to balance the sample size after feature selection. SMOTE oversamples the minority class and undersamples the majority class, as applied in previous studies [33, 48].

Certainly, there are several limitations in this study. First, some patients were excluded from the study cohort for various reasons, so there may be some inevitable selection bias. Second, the two groups of meningioma patients were significantly heterogeneous, which could have affected the predictive performance. Furthermore, all patients were from a single center and no external validation was performed. The performance of our model will vary in other centers using different protocols; thus, prospective cohorts from different centers will be needed in future work. Fourth, we only analyzed preoperative MRI and blood indicators of patients, and combined multiomics studies, including genomics and metabolomics, may achieve better diagnostic performance. Finally, the biological significance of high-dimensional data based on radiomics features must be further elucidated.

In conclusion, the nomogram comprising the radiomics score, PEV, and gender had excellent diagnostic performance in identifying high-grade and low-grade meningiomas. We will evaluate and compare more classification algorithms for a more precise preoperative prediction of meningioma grade in subsequent studies.

### Supplementary Information

Below is the link to the electronic supplementary material.

Supplementary file1 (PDF 277 KB)

Supplementary file2 (XLSX 22 KB)

Supplementary file3 (XLSX 612 KB)

Supplementary file4 (XLSX 69 KB)

Supplementary file5 (XLSX 24 KB)

## Abbreviations

AUC: Area under the curve
BTI: Brain-to-tumor interface
CE-T1: Contrast-enhanced T1-weighted
CNS: Central nervous system
DCA: Decision curve analysis
ICC: Inter-observer correlation coefficient
IQR: Interquartile range
LASSO: Least absolute shrinkage and selection operator
MRI: Magnetic resonance imaging
NPV: Negative predictive value
PPV: Positive predictive value
ROC: Receiver operating characteristic
ROI: Region of interest
SD: Standard deviation
SMOTE: Synthetic minority oversampling technique
SVM: Support vector machine
T2: T2-weighted

## Declarations

### Guarantor

The scientific guarantor of this publication is Hongyang Zhao, who is the corresponding author of the article.

### Statistics and biometry

Zhen Zhao has significant statistical expertise, who is the first author of this article.

### Study subjects or cohorts overlap

No study subjects or cohorts have been previously reported.

### Methodology

• Retrospective

• Case–control study

• Performed at one institution
