# Supplementary material for: Multi-parametric MRI-based machine learning model for prediction of WHO grading in patients with meningiomas
Source: Eur Radiol. 2023 Oct 9;34(4):2468–79. doi: 10.1007/s00330-023-10252-8 (PMC10957672; doi:10.1007/s00330-023-10252-8)

Multi-parametric MRI-based machine learning model for prediction of WHO grading in patients with meningiomas

Electronic Supplementary Material

Supplementary Material 1

NLR= neutrophil-to-lymphocyte ratio
NPR=neutrophil-to-platelet ratio
dNLR=derived NLR= neutrophil/(leukocyte- neutrophil)
LMR=lymphocyte-to-monocyte ratio
MLR=monocyte-to-lymphocyte ratio
PLR=platelet-to-lymphocyte ratio
NPI (prognostic nutritional index) = albumin+(5* lymphocyte)
SII (systemic immune inflammatory index) = platelet count *NLR

The units of the relevant variables are as follows:
Neutrophil: (10^9/L)
Lymphocyte: (10^9/L)
Monocyte: (10^9/L)
Platelet: (10^9/L)
Albumin: (g/L)

Supplementary Material 2

The sequence parameters on the CE-T1 weighted images were as follows: The repetition time (TR) / echo time (TE)= 500/8.4 ms, data matrix = 512 × 512, slice thickness = 5 mm, flip angle = 90◦. The contrast-enhanced scanning was conducted within 200 s after injection of gadopentetate dimeglumine (0.1 mmol/kg). In our study, CE-T1 and T2-weighted images were used for analysis.

The sequence parameters on the T2 weighted images were as follows: TR / TE= 9000/105 ms, data matrix = 512 × 512, slice thickness = 5 mm, flip angle = 90◦.

Supplementary Material 3

The specific algorithm used for tumor volume calculation was as follows:

$$V_i = \frac{O_{a_i} \times (O_{b_i} \times O_{c_i})}{6}$$

Equation (1)

$$V = \sum_{i=1}^{N_f} V_i$$

Equation (2)

where, N_f represents the number of faces (i.e., triangles) defining the mesh, and V is the volume of the mesh in millimeters cubed (mm^3). For each face, i, in the mesh, defined by points a_i, b_i, and c_i, the (signed) volume V_i of the tetrahedron defined by that face, and the origin of the image (O) was calculated using Equation (1). The sign of the volume was determined by the sign of the norm, which must be consistently defined as either facing outward or inward of the ROI. By taking the sum of all V_i values, the total volume of the ROI V was obtained using Equation (2). Volume calculation was conducted using PyRadiomics and Python (version Anaconda 5.0.1; Anaconda, Inc.).

Supplementary Material 4

The mathematical formula of Lasso was as follows:

$$J = \frac{1}{2n} \sum_{i=1}^{n} (y_i - \sum_{j=1}^{p} x_{ij} \beta_j)^2 + \lambda \sum_{j=1}^{p} |\beta_j|$$

Where n is the number of samples, p is the number of features, and y_i is the target variable value of the i sample, x_{ij} is the j th eigenvalue of the i-th sample, β_j is the coefficient of the j feature.

$\lambda \sum_{j=1}^{p} |\beta_j|$ is a regularization item, λ is the weight of the regularization term, which is used to control the goal of the balance fitting training and keep the parameter value to the minimum.

It can be seen that the above formula is the sum of two parts, the first part is the Residual Sum of Squares (RSS), and the second part is the penalty term, which is adjusted by hyperparameter λ identified through cross validation. When certain variables are included in Lasso, it can be ignored that the decrease of RSS value is small, whereas the impact of shrinkage penalty will increase. This means that the coefficient of this variable is zero.

Supplementary Figures

Figure S1. The LASSO regression was performed to minimize the risk of overfitting with the minimum criteria.

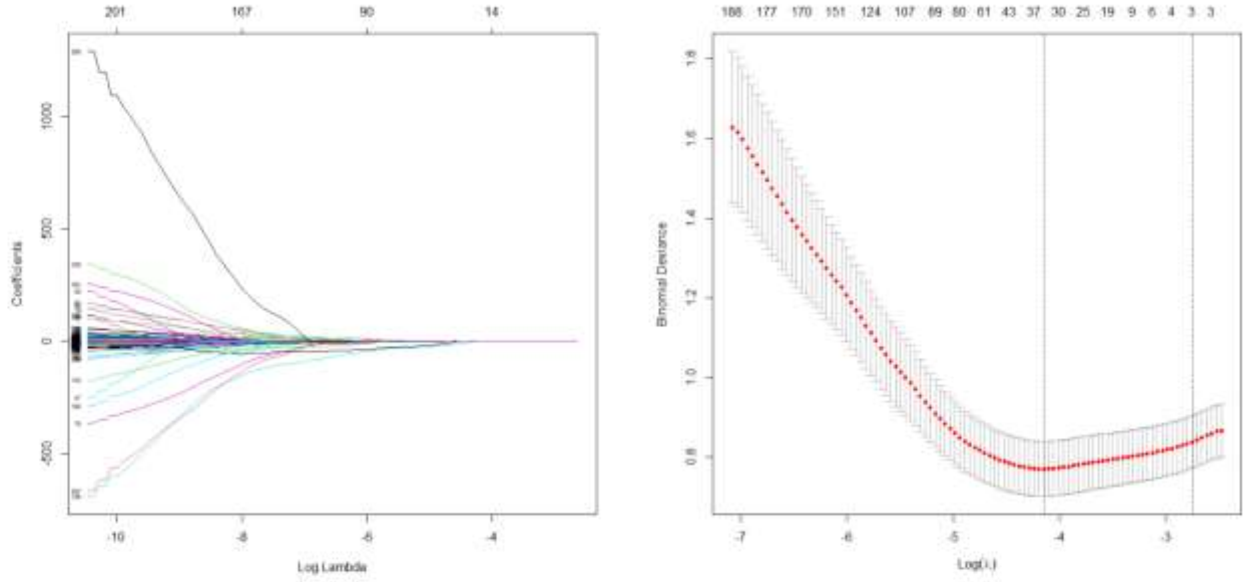

Figure S2. Differential distribution of clinical features in low-grade and high-grade meningiomas.

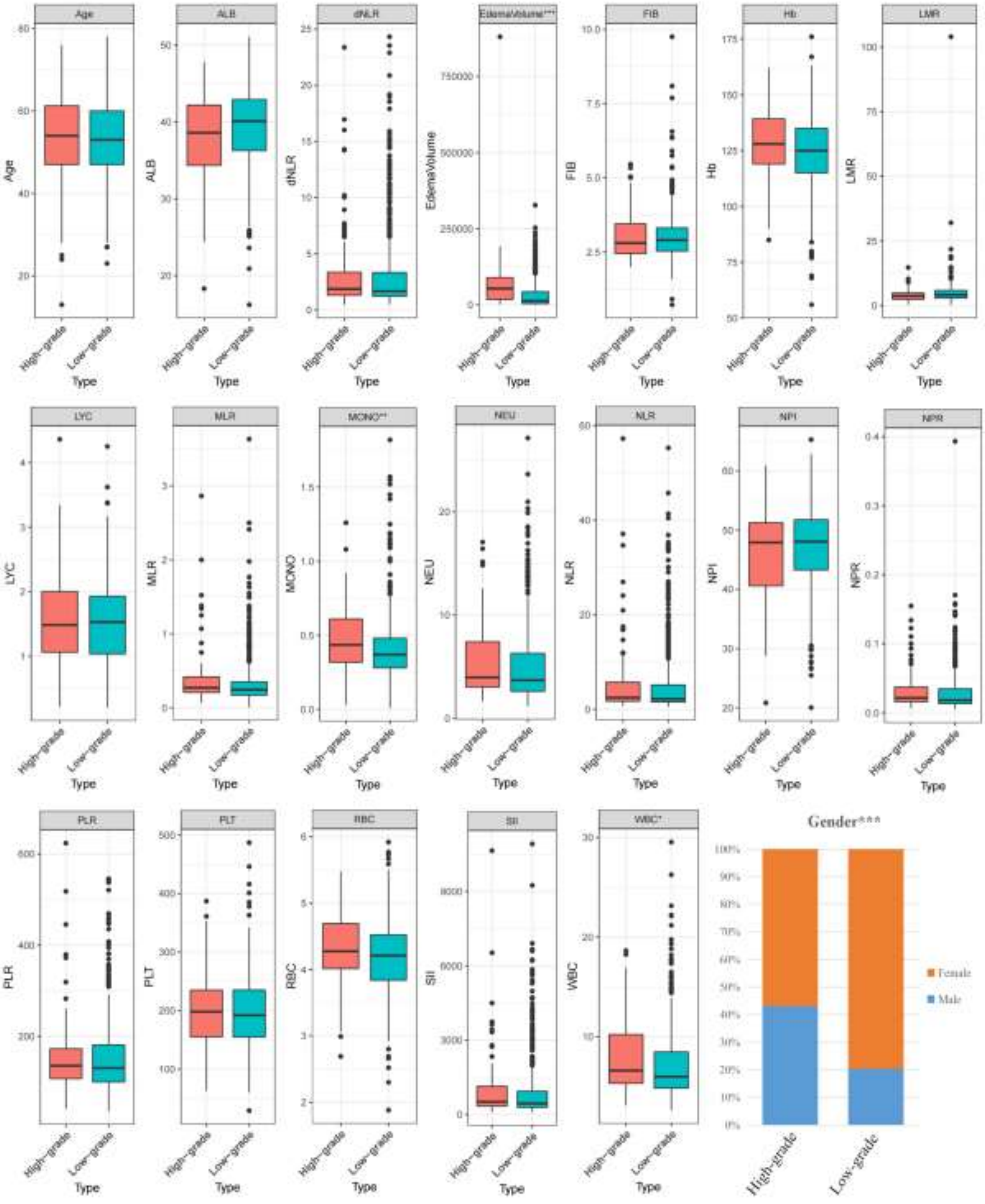

* P < 0.05, ** P < 0.01, *** P < 0.001, and ns No significance.

Figure S3. The heatmap of seven different radiomics signature. The radiomics features were significantly different in the distribution of low-grade and high-grade meningiomas.

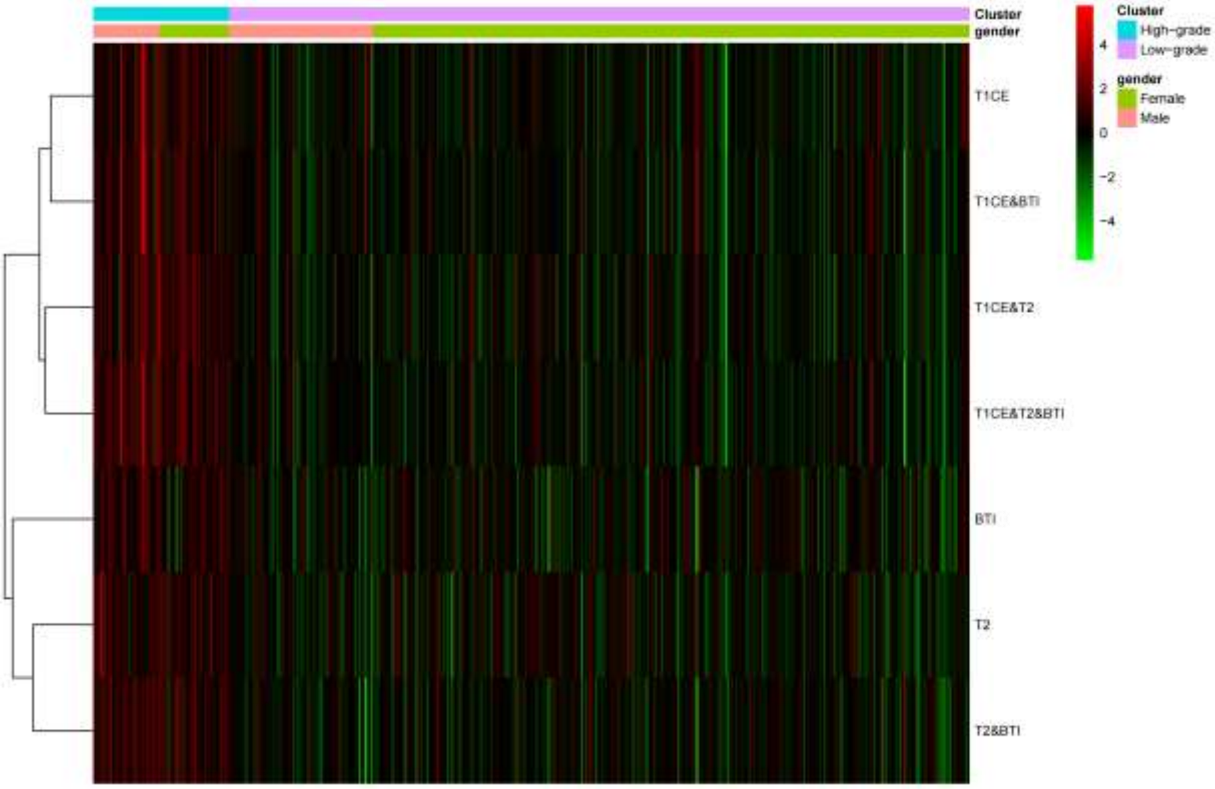

Supplement: Supplementary file 1 — Supplementary file1 (PDF 277 KB) [file 330_2023_10252_MOESM1_ESM.pdf]
